# Role and Function of Mesenchymal Stem Cells on Fibroblast in Cutaneous Wound Healing

**DOI:** 10.3390/biomedicines10061391

**Published:** 2022-06-12

**Authors:** Kotaro Tanaka, Ryohei Ogino, Sho Yamakawa, Shota Suda, Kenji Hayashida

**Affiliations:** 1 Division of Plastic and Reconstructive Surgery, Faculty of Medicine, Shimane University, 89-1 Enya-cho, Izumo 693-8501, Japan; hhelibebcnofne1@icloud.com (K.T.); syama8@med.shimane-u.ac.jp (S.Y.); s.suda@med.shimane-u.ac.jp (S.S.); 2 Department of Life Science, Faculty of Medicine, Shimane University, 89-1 Enya-cho, Izumo 693-8501, Japan; 3 Fukuoka Tokushukai Hospital, 4-5 Sugukita, Kasuga 816-0864, Japan; 4 Department of Frontier Science for Pharmacotherapy, Graduate School of Biomedical and Health Sciences, Hiroshima University, 1-2-3 Kasumi, Minami-ku, Hiroshima 734-8553, Japan; ryogino@hiroshima-u.ac.jp

**Keywords:** mesenchymal stem cell, wound healing, exosome, regenerative medicine, fibroblast

## Abstract

Skin wounds often repair themselves completely over time; however, this is true only for healthy individuals. Although various studies are being conducted to improve wound-healing therapy outcomes, the mechanisms of wound healing and regeneration are not completely understood yet. In recent years, mesenchymal stem cells (MSCs) have been reported to contribute significantly to wound healing and regeneration. Understanding the function of MSCs will help to elucidate the fundamentals of wound healing. MSCs are multipotent stem cells that are used in regenerative medicine for their ability to self-renew and differentiate into bone, fat, and cartilage, with few ethical problems associated with cell harvesting. Additionally, they have anti-inflammatory and immunomodulatory properties and antifibrotic effects via paracrine signaling, and many studies have been conducted to use them to treat graft-versus-host disease, inflammatory bowel disease, and intractable cutaneous wounds. Many substances derived from MSCs are involved in the wound-healing process, and specific cascades and pathways have been elucidated. This review aims to explain the fundamental role of MSCs in wound healing and the effects of MSCs on fibroblasts.

## 1. Introduction

Mesenchymal stem cells (MSCs) are self-renewing multipotent tissue stem cells. MSCs renew themselves and differentiate into bone [1,2], cartilage [3,4], and fat [5,6], which in turn transform into cells that constitute each of these tissues. MSCs can be harvested from bone marrow [7,8], adipose tissue [9,10], placenta [11,12], and dental pulp [13,14]. On the other hand, embryonic stem (ES) cells are pluripotent stem cells derived from the inner cell mass of a blastocyst, an early-stage pre-implantation embryo. ES cells for clinical use have always raised ethical issues. However, researchers and physicians can avoid ethical problems in MSCs transplantation because MSCs can be harvested and cultured from adult tissues. They are one of the stem cells that are expected to be applied in regenerative medicine. One of the classes of cells that MSCs can differentiate into is fibroblasts [15]. Fibroblasts participate in the structure of all tissues in the body (skin, muscle, nerve, etc.) and are the primary cells of the connective tissue. Furthermore, fibroblasts not only constitute tissues but are also involved in the proliferation of cancer cells [16,17]. Thus, fibroblasts are essential for the maintenance of human life and are also involved in the malignant transformation of cells. Fibroblasts are also important cells for both clinical and academic purposes. In skin wound healing, fibroblasts play a major role in damage repair, and successful control of fibroblast collagen production not only promotes wound healing but also prevents scar formation [18,19,20]. The promotion of skin wound healing has been extensively studied as a form of regenerative medicine using MSCs [21,22]. Reportedly, MSCs migrate and proliferate in the damaged area to fill in the defect and properly manage fibroblast proliferation and collagen production. This review summarizes the basic description of MSCs as a regenerative medicine, the interaction between MSCs and fibroblasts, how the two cells interact in wound healing, and relevant problems.

## 2. Cutaneous Wound-Healing Process

The skin wound-healing process consists of three phases: inflammation, proliferation, and remodeling [23]. When external stimuli are applied to the skin causing damage, the skin tissue breaks down, and its destruction occurs. Simultaneously, blood vessels in the path of the injury are severed, causing blood to leak [24]. Platelets in the blood adhere to damaged and exposed collagen fibers and get activated [25]. Activated platelets subsequently activate coagulation factors, which convert prothrombin to thrombin [26,27], which in turn converts fibrinogen to fibrin [28]. Fibrin engulfs platelets and red blood cells to form clots. This series of events is the hemostatic effect of the blood coagulation reaction. In addition to clotting factors, various chemicals are released into the blood that leaks out due to vascular damage [29]. Additionally, cell membranes of the destroyed cells undergo chain reactions in the arachidonic acid cascade [30], releasing a variety of chemicals. These released chemicals create gaps in the endothelial cells of the capillaries and dilate them, allowing spherical polymorphonuclear leukocytes and mononuclear cells to escape from the vessels as effusions and migrate toward the wound [31]. At this time, proinflammatory cytokines are released by the phagocytosis of mononuclear cells. This induces four clinical symptoms: redness, swelling, pain, and fever. In the second proliferative phase, the released inflammatory cytokines cause fibroblasts to migrate to the wound and produce collagen fibers [32]. They also act on the endothelial cells of the capillaries to promote angiogenesis [33]. The newly created blood vessels supply the wound with blood, which in turn supplies fibroblasts with the oxygen and nutrients they need for proliferation. Through this sequence of events, the skin defect is filled, and the wound heals. The tissue that fills these defects is called the granulation tissue, and by combining with substances other than collagen, it transforms into a solid tissue such as the dermis. At this stage, it is called scar tissue [18,34,35]. In the final stage of skin wound healing, fibroblast function and collagen production decrease, but the amount of collagen produced is equal to the amount degraded and absorbed. Although the tissue formed in this manner appears to be unchanged, it is an active tissue that is constantly being produced and broken down. The reconstructed subepidermal tissue does not replace the dermis but remains a permanent scar tissue [19]. The epidermis looks almost the same as before the injury, but the scar tissue looks somewhat different from the dermis because of the irregular arrangement of collagen. This is called a scar. Once scar tissue matures, the surrounding tissue cannot regenerate normal dermal tissue [21,22,36]. Therefore, one of the challenges of wound healing is not to eliminate scar tissue but to reduce the amount of scar tissue and make it resemble the normal dermis. In recent years, MSCs have been widely applied to promote wound healing [37]. The treatment and prevention of aberrant scarring using MSCs have been studied extensively in basic studies but are not yet in clinical applications.

## 3. Mesenchymal Stem Cells (MSCs)

MSCs were first isolated from bone marrow in 1960–1970 and were characterized as cells with fibroblastoid shape and osteogenic potential, which form clonogenic colonies [22]. Depending on their tissue of origin, MSCs exhibit different characteristics. They are stem cells that can self-renew and proliferate [38] and differentiate into fat [5,6], bone [1,2], and/or cartilage [3,4] in the human body. They are present in all tissues and can be harvested from the placenta [11,12], bone marrow [7,8], fat tissues [9,10], and dental pulp [13,14]. MSCs reside in a non-hematopoietic population and can be cultured to form fibroblast-like colonies in vitro [39,40]. They are expected to make a major contribution to regenerative medicine because they can be isolated using a relatively simple technique [41,42] and because there are very few ethical issues, they can be easily applied to clinical trials [43]. There have been over 1000 clinical trials exists with MSCs over 25 years [44], and their numbers are growing day by day. MSCs are being studied for the treatment of fractures [45,46], arthritis [47], osteonecrosis of the jaw [48,49,50], graft-versus-host disease [51], Crohn’s disease [52], multiple sclerosis [53], stroke [54], myocardial infarction [55], liver cirrhosis [56], and chronic lung disease [57]. Furthermore, they have immunosuppressive functions, and many studies are underway to develop therapies that take advantage of this function. For that reason, MSCs have been reported to be particularly effective in the treatment of inflammatory bowel disease [58] and autoimmune disorders [59]. However, there are some issues regarding the methods of isolation and collection of MSCs. The MSCs that are currently available are obtained by seeding bone marrow mononuclear cells on culture dishes and collecting attached cells after 2–3 weeks of culture [7]. Regarding differentiation potential, the presence of hematopoietic cells and foreign cells with low or completely lost differentiation potential is inevitable, and undifferentiated cells with actual differentiation and proliferation potential (high-quality MSCs) are present only at a low frequency [60]. Regarding proliferative ability, MSCs, which are primary cultured cells, have a limited number of proliferation cycles and usually lose proliferative potential after 3–5 passages. At this time, their differentiation potential was significantly reduced. In addition, MSCs cannot be used for the treatment of systemic diseases by intravenous administration because the cells transform during culture amplification. When MSCs pass through the blood vessels of the lungs, they should be trapped in the small capillaries of the lungs [61,62]. Therefore, for safe and effective cell therapy, it is necessary to guarantee cellular functions of the MSCs, such as proliferation and differentiation capacity, as well as cell uniformity and migration capacity. Many studies have been conducted to resolve these problems, to recognize proteins expressed on the cell surface of MSCs as antigenic markers, and to isolate high-quality MSCs by analyzing their expression patterns using several types of markers [63]. Therefore, the collection of high-purity, higher-quality MSCs and the management of cultured MSCs for their use in regenerative medicine is a problem that needs to be solved as soon as possible [64,65]. Although MSCs can be used for many diseases and have significant advantages, there still are issues in establishing collection methods in clinical settings.

### 3.1. Bone Marrow-Derived MSCs

Bone marrow has long been used to harvest MSCs in vivo. In general, when MSCs are used to promote wound healing in basic experiments and clinical applications, they are most likely bone marrow-derived. Therefore, the characteristics of bone marrow-derived MSCs should be known and described first. The International Society for Cellular Therapy proposed the following criteria for the identification of MSCs: adherence to plastic; differentiation into chondrocytes, osteoblasts, and adipocytes under standard in vitro differentiation conditions; and expression of surface markers CD105, CD73, and CD90, in the absence of CD45, CD34, CD14, CD11b, CD79α, CD19, and HLA-DR [66]. MSCs, particularly bone marrow-derived MSCs, are found in a variety of tissues in vivo and have been extensively studied [67]. MSCs in human bone marrow were initially thought to constitute the microenvironment of hematopoietic stem cells (HSCs) and play a role in maintaining tissue homeostasis [68]. The bone marrow niche is a vital microenvironment that regulates many stem cell activities, including self-renewal, mobilization, engraftment, and lineage differentiation [69]. Niches are the local tissue microenvironments that maintain and regulate stem cells. Hematopoiesis provides a paradigm for understanding mammalian stem cells and their niches; however, the HSC niche remains incompletely defined and beset by competing models [70]. Bone marrow-derived MSCs lose their proliferative, differentiation, and migratory abilities with age because the bone marrow changes from red marrow to fat marrow with age in humans. As MSCs age, their cell size increases, and they eventually undergo apoptosis and die [71]. These characteristics have been reported to have similar results in mouse MSCs as well as human MSCs [72]. MSC fractions were collected from bone marrow cells by measuring the expression pattern of proteins on the cell surface and the fluorescence intensity by flow cytometry. Various MSC fractions have been defined from the bone marrow, and the expression patterns of markers have been investigated, but the question remains as to which is the true MSC fraction. It is assumed that all fractions contain at least some true MSCs, but a large percentage of cells other than MSCs (erythrocytes, mononuclear cells, and fibroblasts) are thought to be present. For example, if the MSC fraction contains a high proportion of fibroblasts, cells taken and cultured from the bone marrow would be fibroblasts rather than MSCs and would not differentiate into bone or fat. Thus, MSCs and fibroblasts are largely related, and there is an urgent need to develop a method that can completely distinguish between MSC and fibroblasts. Sudo et al. considered various primary fibroblast-like cultures and showed that all cells studied expressed CD44, CD90, and CD105, whereas none of them expressed CD14, CD34, and CD45 [73]. Although fibroblasts are increasingly being reported to have an MSC-like cell surface immunophenotype (cell surface protein), MSCs have also been perceived to be positive for fibroblast markers such as collagen, vimentin, fibroblast surface protein, heat shock protein 47 (HSP47), and α smooth muscle actin (αSMA) [74,75]. Although several markers have been identified for the two cell types, one of the key problems associated with the proper characterization of both cell types is that they are generally nonspecific. The two types of cells might be categorized by a combination of nonspecific markers rather than a few specific markers. There are also many conflicting results regarding the characterization of cell surface markers on MSCs and differences between MSCs and fibroblasts. The variability in results may be due to a variety of factors, including differences in the tissue of origin, donor heterogeneity, and lack of adequate studies to specifically identify differences between these two related cell types [76]. Thus, MSCs harvested from bone marrow have great potential for clinical application owing to their high proliferative and differentiation potential, but problems arise when harvesting bone marrow from a living person because of the small quantity extracted and the labor-intensive nature of the process. In most cases, bone marrow is harvested from the iliac bone, from which MSCs are isolated and cultured. However, because only a small number of bone marrow cells can be obtained from a single bone marrow biopsy, the number of MSCs that retain multilineage differentiation potential is small. In addition, the technology and environment for culturing and managing MSCs in a completely clean environment are required to increase their numbers, which is time-consuming and costly. In addition, the risk of side effects from bone marrow biopsy must be considered. 

### 3.2. Adipose Tissue-Derived MSCs 

Understanding adipose-derived MSCs compared to bone marrow-derived MSCs also provides a deeper understanding of the characteristics of MSCs. In particular, plastic surgeons involved in wound healing will be able to obtain adipose-derived MSCs through liposuction procedures; therefore, research is expected to advance further in the future. As adipose tissue is one of the factors contributing to obesity, and many people may not have a suitable perception of fat. Liposuction is becoming increasingly common worldwide and a common procedure in the field of plastic surgery [77,78]. In addition, in regenerative medicine, subcutaneous adipose tissue is attracting attention as a new tissue stem cell source to replace bone marrow because it can be harvested in large volumes, has a high percentage of multipotent cells, can be easily cultured in large volumes, and has characteristics similar to those of bone marrow-derived MSCs [79]. Although these are few reports, adipose tissue-derived MSCs can differentiate into numerous cell forms such as osteocytes, adipocytes, neural precursor cells, vascular endothelial cells, myocytes, pancreatic β cells, and hepatocytes [80,81,82,83,84]. Human adipose tissue-derived MSCs express conventional MSC surface markers, including the cell adhesion molecules CD29, CD44, CD146, and CD166, the receptor molecules CD90 and CD105, and the GPI-anchored enzyme CD73. Furthermore, adipose tissue-derived MSCs should be negative for hematopoietic cell surface antigens, including CD11b, CD13, CD14, CD19, and CD45. They are also negative for endothelial markers CD31 and HLA-DR [85]. From a clinical standpoint, adipose tissue-derived MSCs and bone marrow-derived MSCs are considered to have similar capabilities with some molecular differences (immunophenotype, differentiation potential, transcriptome, proteome, and immunomodulatory activity). Furthermore, adipose tissue-derived MSCs may be more suitable for the treatment of several diseases than bone marrow-derived MSCs because of their less invasive and safe technique [86]. Bone marrow was the first tissue used in vivo for MSC collection; however, in recent years, adipose tissue has been used more frequently because of its simplicity. Adipose tissue-derived MSCs are more easily obtained from the donor sites than bone marrow-derived MSCs and are extensively proliferative ex vivo and in vitro. However, the question arises as to whether MSCs derived from adipose tissue are fully immature and undifferentiated. If MSCs in adipose tissue are adipocyte-prone MSCs, they may revert from adipocytes to a more undifferentiated state as MSCs. The mechanisms of maintenance of MSC multipotency are still unrevealed. We believe that the differences in MSC characteristics are affected by the differences in their origin. Further research is needed to investigate the characteristic of MSCs multipotency and apply them to regenerative medicine. Specifically, MSCs derived from adipose tissue increased renal blood flow and improved renal function when administered to patients with focal segmental glomerulosclerosis [87]. The in vivo kinetics of the MSCs used in this clinical trial have not been elucidated, and the mechanism may be investigated using gene modification techniques.

### 3.3. MSCs Promote Wound Healing 

**Figure 1:**
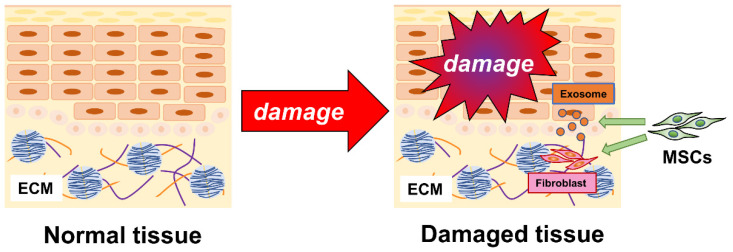
Mesenchymal stem cells promote cutaneous wound healing.

Understanding the interaction between MSCs and wound healing is important because we believe that understanding this interaction can help prevent scarring and promote reparative healing. The mechanisms by which MSCs treatments promote wound healing include secretion of angiogenesis-promoting factors, differentiation into wound-healing cells such as fibroblasts, mobilization of intrinsic stem cells, generation and remodeling of the extracellular matrix, the polarization of M2 macrophages, and immunosuppressive effects. MSCs in the stem cell niche are known to repair damaged or dead cells by proliferating and differentiating into skin cells, but they also activate cell recovery and healing processes through an autocrine and paracrine pathway [88]. In addition, cell-cell interactions between MSCs and macrophages influence skin wound healing during MSC transplantation, but the detailed mechanisms are still unclear [89]. Exosomes play an important role in paracrine functions and are involved in wound healing by MSCs. MSC-derived exosomes have attracted attention not only for wound healing but also for their immunomodulatory functions [90]. Many studies have confirmed the anti-inflammatory [91] and wound-healing effects of MSC exosomes under various conditions. MSC exosomes may be an excellent alternative to MSC cell therapy because they are more stable and less immunogenic than derived cells while having the same biological functions as derived cells [92,93]. The use of MSC-derived exosomes is being investigated as a possible treatment modality for future clinical applications. Current research on MSC exosomes in wound healing and skin regeneration has also focused on the role of MSC exosomes in the three phases (inflammation, proliferation, and remodeling) [94]. Prolonged inflammation delays wound healing and cause scarring. Therefore, it is important to control inflammation and the transition to the proliferative phase [95]. During the inflammatory phase of the wound-healing process, MSC-derived exosomes can change the polarity of M1 macrophages to M2 macrophages, which in turn have been reported to have anti-inflammatory and immunomodulatory properties that promote wound healing [96,97]. Induced M2 macrophages secrete anti-inflammatory factors, such as IL-10 and transforming growth factor-β (TGF-β), and M2 markers, such as IL-1RA, CD163, and C-C motif chemokine 22 (CCL22) [98]. These actions are important for wound repair. These actions suppress inflammation and promote wound healing. Second, both MSC exosomes and MSCs play a role in the proliferative phase. MSC-derived exosomes enhance the proliferation and migration of human keratinocytes and dermal fibroblasts [99,100]. In addition, MSC has been shown to migrate into wounds and differentiate into fibroblasts, keratinocytes, and other wound-healing cells in the skin [15]. Finally, in the last remodeling phase, the extracellular matrix composed of type III collagen is converted to type I collagen [101]. Dermal fibroblasts produce collagen and extracellular matrix, which are ultimately important in tissue remodeling. However, if fibroblasts fail to function properly and produce excessive collagen fibers, they may eventually cause scarring [20]. The antifibrotic effects and regulation of collagen-producing fibroblast function of MSC-derived exosomes are important for the prevention of scarring, but they are not well understood. Although this review discusses MSCs and fibrosis with a focus on skin wound healing, treatments for fibrosis in other organs have also been developed using the antifibrotic properties of MSCs [102]. The extent to which MSCs influence fibroblasts during wound healing is also important (Figure 1).

When normal skin tissue is damaged by external factors, resident and/or infused MSCs migrate to the damaged area [88,103]. The migrating MSCs act on fibroblasts in the dermis to stimulate the production of appropriate amounts of collagen fibers to repair the damaged area. In addition, exosomes secreted by MSCs prevent scar formation and help the skin to return to tissue similar to normal skin tissue.

### 3.4. MSCs and Fibroblasts 

MSCs are closely related to fibroblasts during the wound-healing process, and appropriate proliferation control is necessary because collagen regeneration by fibroblasts, especially during the proliferative and remodeling phases, can cause scar formation [20]. Fibroblasts are cells present in all tissues in the body and are largely responsible for the production of the extracellular matrix. Human bone marrow-derived MSCs express CD90, and fibroblasts express CD90′ detected in many tissues, including the human myometrium, orbit, and lung. As both cells express CD90, analysis of the expression pattern of the antigen on the surface of each cell is important to distinguish between the two. Adult CD90-positive fibroblasts exhibit stem cell characteristics and high immaturity potential. These cells are initially isolated and cultured and have the potential to differentiate into other cell types [104]. Furthermore, fibroblasts have anti-inflammatory, immunomodulatory, and regenerative properties similar to MSCs, and their application in regenerative medicine has been discussed [105,106]. It has been reported that MSCs and fibroblasts are deeply involved via macrophages and exosomes not only in wound healing and regenerative medicine but also in the field of cancer [16,17]. As the similarity in location and cell characteristics between MSCs and fibroblasts, the problem with conventional MSC isolation methods is that both types of cells can be confused. The conventional method was to isolate only whole cells from bone marrow, adipose tissue, dental pulp, and other tissues and then sow cells in flasks and culture cells to form colonies, which were then used as MSCs. However, this method cannot isolate pure MSCs, because fibroblasts also form colonies. Therefore, a more efficient MSC isolation method is required. Recently, exosomes have attracted attention in the interaction between MSCs and fibroblasts, and cell-free therapy using MSC-derived exosomes instead of MSCs has been investigated [107,108]. Several studies about MSCs’ function on fibroblasts are summarized in Table 1. Molecular biology elucidation of the interaction between MSCs and fibroblasts will provide more treatment options in the future.

### 3.5. MSC-Derived Exosome 

Currently, MSC-derived exosomes are expected to have wound healing and anti-inflammatory effects. MSCs are thought to exert their physiological activity through paracrine action, and exosomes are the transporters responsible for this action. Extracellular vesicles are lipid-binding nanoparticles secreted from the cell into the extracellular space, and the exosome is one of the extracellular vesicles [115]. Exosomes are also released by cells other than MSCs and are responsible for intercellular communication. They are granule-like substances with a diameter of 50–150 nm secreted by cells. Its surface contains lipids and proteins derived from the cell membrane, whereas its interior contains nucleic acids (microRNA, messenger RNA, and DNA), proteins, and other intracellular substances. Vesicles with exosome characteristics can be isolated from fluids, such as semen [116], urine [117], saliva [118], blood [119], bile [120], milk [121], cerebrospinal fluid [122], and ascites [123]. Various tissue-derived MSCs secrete exosomes; however, the function of exosomes differs depending on the tissue of origin. Although only one case has been reported, bioinformatic analysis has revealed that exosomes from bone marrow-derived MSCs have a high regenerative capacity, those from adipose tissue-derived MSCs have a high immunomodulatory capacity, and those from umbilical cord-derived MSCs have high tissue damage repair capacity [124]. Bone marrow-derived MSCs exosomes can be used to treat kidney disease [125]. Exosomes from adipose tissue-derived MSCs are effective in healing skin injury, nerve regeneration, ischemia-reperfusion, parenchymal organ diseases, and obesity. In particular, adipose tissue-derived MSCs can increase type I and type III collagen production via the PI3K/Akt signaling pathway in fibroblasts. Furthermore, exosomes from adipose-derived MSCs have been found to prevent scarring, suggesting that cell-free therapy is an effective strategy [126]. Umbilical cord-derived MSCs exosomes can be used to treat liver fibrosis [127]. As the effect and mechanism of action of MSC exosomes vary depending on the tissue from which they are derived, the diseases and molecules targeted for treatment will naturally differ. For example, in an in vitro model of Alzheimer’s disease, adipose tissue-derived MSC exosomes appeared to be more effective in degrading Aβ than bone marrow-derived MSC exosomes [128]. When discussing the therapeutic effects of MSC-derived exosomes in the future, it is necessary to consider which tissues they are derived from and what mechanism of action is expected. An additional therapeutic approach that focuses on the benefits of other MSC-derived exosomes is cell-free therapy. Cell-free therapy using MSC-derived exosomes has attracted attention owing to its various advantages. In contrast to therapies that use whole cells, cell-free therapies that use MSC-derived exosomes are easier to manage and safer because they contain lower amounts of membrane-bound proteins, such as MHC molecules, and do not directly form tumors. Cell-free therapy using MSC-derived exosomes has been reported to be effective in wound healing, lung cancer, cardiovascular disease, and COVID-19 [129]. Thus, cell-free therapy is currently attracting attention as a regenerative therapy in which MSC-derived exosomes will play an important role.

### 3.6. Future Prospects of MSCs

**Figure 2:**
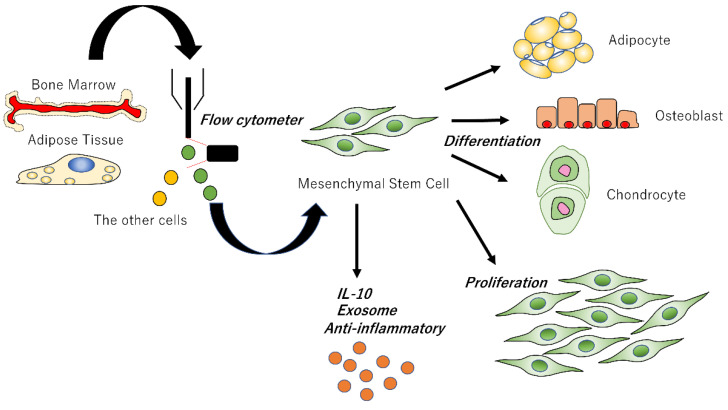
Isolation and differentiation of high-grade mesenchymal stem cells.

Currently, research institutes and companies are developing methods for the mass culture of high-quality MSCs, but there are very few successful examples, and they cannot be applied in clinical settings. Therefore, researchers and physicians are carefully culturing flasks one by one and administering them to individual patients. The establishment of a simple and accurate mass culture method for MSCs is one of the issues that must be solved immediately. Cell sorting by flow cytometer needs not necessarily be used to harvest and isolate MSCs. However, we prefer to use a flow cytometer to collect highly pure MSCs efficiently. Crisan M et al. investigated the expression of MSC markers detected at the surface of native, non-cultured perivascular cells using a flow cytometer [130]. Although the use of a flow cytometer may have additional disadvantages in terms of cost and time, it should be useful in elucidating MSCs and further development. (Figure 2). Even when used for wound healing, MSCs will be able to be administered via intravenous infusion or topical injection without culture simultaneously [131]. A difficult administration method will cause confusion in the clinical setting and itself makes it difficult to further development. Ideally, MSCs should be administered in a manner that promotes rapid wound repair, similar to the administration of antimicrobial agents. Furthermore, it is hoped that the exosomes and wound-healing substances secreted by MSCs could be used intravenous injection instead of using MSCs in themselves, and these substances could provide to patients more conveniently due to the development of self-administration drugs. 

To collect genuine MSCs, MSCs can be harvested and isolated from bone marrow and adipose tissue using a flow cytometer. Isolated MSCs can differentiate into osteocytes, adipocytes, and chondrocytes for forming the organization or self-renew to further increase the number of MSCs. MSCs themselves also secrete anti-inflammatory substances such as IL-10 and secrete exosomes to produce paracrine action.

## 4. Conclusions

Accelerated wound healing and proper collagen production by fibroblasts is a very important process as it prevents scar formation. MSCs have long been used in regenerative medicine owing to fewer ethical issues, relatively easy to harvest from organisms, a small risk of immune rejection if self-derived MSCs are used, smaller risk of tumor formation compared to induced pluripotent stem cells and ES cells. In recent years, MSC-derived exosomes have attracted attention for their inflammation-modulating properties, and their application in wound healing is expected. Since MSCs and fibroblasts are closely interrelated, fundamental elucidation of these mechanisms will greatly contribute to regenerative medicine, especially in plastic surgery and dermatology. However, some reports are skeptical about whether MSCs act on fibroblasts in the skin or affect wound healing [131,132,133,134]. The significance of using stem cells for chronic wound healing has been discussed, and there is a growing understanding of the mechanisms and pathophysiology.

## Figures and Tables

**Table 1:** MSC functions on fibroblast under different conditions.

Experiment Situation	Biological Signals	Effects	Ref.
Human bone marrow-derived MSC-CM in wound healing in DM	EGF, bFGF	Increase proliferation, cell viability, and migration of fibroblast	[109]
EGF transferred umbilical cord blood-derived MSC	β-catenin, N-cadherin, cofilin, ezrin, phospho-MAPK/CDK substrate, phospho-Arg-(Ser)-X-Tyr/Phe-X-pSer motif	Increase cell adhesion, dynamic effects, migration, and proliferation of fibroblast	[110]
Adipose-derived MSC in wound healing	TGF-β	Increase collagen production and inhibit fibroblast proliferation by avoiding excessive fibrogenesis	[111]
Human ES cell-derived endothelial precursor cells in cutaneous excisional wound models	EGF, bFGF	Increase proliferation and migration of fibroblast	[112]
Human amniotic mesenchymal stem cells	LOXL2	LOXL2 significantly enhanced in vitro keratinocyte migration and differentiation	[113]
Human umbilical cord Wharton’s jelly-derived MSC in DM	Cytokeratin, involucrin, filaggrin, ICAM-1, TIMP-1, and VEGF-A	Increase the number of invaded cells, cell viability, total collagen, elastin, and fibronectin levels	[114]

Abbreviations: bFGF, basic fibroblast growth factor; CDK, cyclin-dependent kinase; CM, conditioned medium; DM, diabetes mellitus; EGF, epidermal growth factor; ES, embryonic stem; ICAM-1, intercellular adhesion molecule 1; LOXL2, lysyl oxidase-like 2; MAPK, mitogen-activated protein kinase; MSC, mesenchymal stem cell; TGF-β, transforming growth factor-β; TIMP-1, tissue inhibitors of metalloproteinases-1; VEGF-A, vascular endothelial growth factor-A.
